# *Candida albicans* Yeast, Pseudohyphal, and Hyphal Morphogenesis Differentially Affects Immune Recognition

**DOI:** 10.3389/fimmu.2017.00629

**Published:** 2017-06-07

**Authors:** Liliane Mukaremera, Keunsook K. Lee, Hector M. Mora-Montes, Neil A. R. Gow

**Affiliations:** ^1^Aberdeen Fungal Group, Institute of Medical Sciences, Foresterhill, University of Aberdeen, Aberdeen, United Kingdom; ^2^Department of Microbiology and Immunology, Medical School, University of Minnesota, Minneapolis, MN, United States; ^3^Departamento de Biología, División de Ciencias Naturales y Exactas, Universidad de Guanajuato, Guanajuato, Mexico

**Keywords:** *Candida albicans*, cell wall, cytokine, immune recognition, morphogenesis

## Abstract

*Candida albicans* is a human opportunist pathogen that can grow as yeast, pseudohyphae, or true hyphae *in vitro* and *in vivo*, depending on environmental conditions. Reversible cellular morphogenesis is an important virulence factor that facilitates invasion of host tissues, escape from phagocytes, and dissemination in the blood stream. The innate immune system is the first line of defense against *C. albicans* infections and is influenced by recognition of wall components that vary in composition in different morphological forms. However, the relationship between cellular morphogenesis and immune recognition of this fungus is not fully understood. We therefore studied various vegetative cell types of *C. albicans*, singly and in combination, to assess the consequences of cellular morphogenesis on selected immune cytokine outputs from human monocytes. Hyphae stimulated proportionally lower levels of certain cytokines from monocytes per unit of cell surface area than yeast cells, but did not suppress cytokine response when copresented with yeast cells. Pseudohyphal cells induced intermediate cytokine responses. Yeast monomorphic mutants had elevated cytokine responses under conditions that otherwise supported filamentous growth and mutants of yeast and hyphal cells that were defective in cell wall mannosylation or lacking certain hypha-specific cell wall proteins could variably unmask or deplete the surface of immunostimulatory ligands. These observations underline the critical importance of *C. albicans* morphology and morphology-associated changes in the cell wall composition that affect both immune recognition and pathogenesis.

## Introduction

Fungal pathogens are associated with a wide range of human diseases from superficial infections of the skin and mucosal surfaces to life-threatening systemic infections, depending on host health and immunocompetence. *Candida* species account collectively for as many as 400,000 cases of systemic fungal disease with associated mortality rates of up to 40% (1–4). Of these species, *Candida albicans* is the most common agent of disease and is characterized by its morphological plasticity. It is capable of vegetative growth *in vitro* and *in vivo* as ovoid budding yeast-like cells and as branching filamentous cells that exist as more or less elongated and constricted chains of yeast cells called pseudohyphae or parallel-sided hyphal cells (5–10). Other cell types, such as GUT, gray, and opaque cells, are a tristable system of specialized cells involved in colonization of specific body sites and in mating competence (10). We set out to characterize differences in the immune response by human peripheral blood mononuclear cells (PBMCs) to yeast cells, hyphae, and pseudohyphae as the three major morphological forms of *C. albicans*.

The innate immune system is the first line of defense against all invading organisms and plays a major role in resistance to infectious diseases in immunocompetent hosts (11). Phagocytes detect microbial pathogen-associated molecular patterns (PAMPs) *via* pattern-recognition receptors (PRRs), resulting in signaling-mediated transcription and secretion of inflammatory mediators, such as chemokines and cytokines that recruit neutrophils and other immune cells to the site of infection, resulting in localized killing of the pathogen and activation of the adaptive immune response (11–13).

*C. albicans* PAMPs that activate the inflammatory response are located in both the outer and inner layers of the intact cell wall (4, 11, 14–16). Mannans and glucans are the main elicitors of both cytokine production and phagocytosis and are recognized by a range of C-type lectins and toll-like receptors (TLRs) (4, 17–21). The *O*-linked mannans are sensed through the TLR4 receptor (17), β-mannan is recognized by galectin-3 (22–25), and α-linked *N*-mannans are recognized by the mannose receptor (MR), dectin-2, mincle, and DC-SIGN (21, 26). Opsonized β1,6-glucan acts as an immune agonist (27), and chitin is taken up by the MR and induces TLR9- and NOD2-dependent IL-10 production (4, 28).

Most immune recognition studies have focused on *C. albicans* yeast cells as the cell target; however, it is known that filamentous hyphal cells induce an altered immune response (4, 6, 8, 21, 29–32). The switch between yeast and hyphal growth is critical for virulence (6, 8, 33, 34), affecting numerous properties including the expression of morphology-dependent cell wall adhesins, invasins, proteases, and a raft of other phenotypic and biochemical properties, including the recently discovered candidalysin toxin (35). Mutants locked in either the yeast or hypha form are avirulent, suggesting that the ability to transit reversibly between these morphotypes potentiate the virulence of this fungus (7, 33, 35–40). Pseudohyphae are a distinct growth form that differs from both yeast cells and parallel-sided hyphae and are characterized by synchronously dividing elongated yeast cells (5, 7, 41, 42). Although pseudohyphal forms are generated by a wide range of *Candida* species, we know little about the immune response to pseudohyphal cells.

It is therefore important to understand the consequences of cellular morphogenesis of *C. albicans* on immune recognition and the activation of inflammation. Here, we demonstrate that *C. albicans* hyphae stimulated lower levels of cytokine production from human PBMCs than did yeast cells, but did not suppress the immune response of yeast cells in trans. Pseudohyphae elicited intermediate cytokine profiles between those of yeast and hyphae and again did not suppress yeast-induced cytokines. We also demonstrate that cell wall mannosylation and certain hypha-specific cell wall proteins affect morphology-dependent recognition by PBMCs.

## Materials and Methods

### Strains, Media, and Culture Conditions Inducing Cellular Morphogenesis

Strains used in this work are listed in Table S1 in Supplementary Material. Cells were maintained and propagated at 30°C in either Sabouraud broth [1% (w/v) mycological peptone, 4% (w/v) glucose] or YPD broth [1% (w/v) yeast extract, 2% (w/v) mycological peptone, 2% (w/v) glucose]. The immune reposes to hyphae induced by multiple independent growth conditions were compared. Hyphae were generated using multiple independent methods: (i) 20% (v/v) fetal calf serum (FCS) or in RPMI 1640 supplemented with 2.5% (v/v) FCS, (ii) in YPD broth supplemented with 20% (v/v) FCS, (iii) in SC broth [0.68% (w/v) yeast nitrogen base without amino acids, 0.074% (w/v) amino acids buffered with 0.378% (w/v) PIPES] supplemented with 0.012% (w/v) fresh *N*-acetylglucosamine (GlcNAc), or (iv) in Lee’s medium (43). Cultures were collected for use when greater 90–95% filamentation was obtained (typically after 3.5 h of incubation at 37°C). Hyphae were then washed twice in PBS and stored frozen at −20°C until used in cytokine induction experiments.

*C. albicans* pseudohyphae were produced using conditions published previously with modifications (41). Overnight cultures of *C. albicans* were collected by centrifugation, washed twice with 0.15 M NaCl, resuspended in 0.15 M NaCl, and incubated at room temperature for 24 h to induce starvation. After 24-h starvation, cells were inoculated into RPMI 1640 at a final concentration of 1 × 10^6^ cells/ml and incubated at 25, 30, or 37°C with shaking for 6 h. Under these conditions, the vegetative morphology could be regulated by growth temperature alone, with yeast cells formed at 25°C, pseudohyphae at 30°C, and true hyphae at 37°C. Heat-killed (HK) cells were prepared after incubation at 56°C for 1 h, with killing verified by plating on YPD.

Samples of cells were fixed in 5% (v/v) formalin for morphological and microscopical analyses. All photomicrographs were taken on an Olympus BX50 outfitted with an Infinity 1 digital camera. Morphology indices, which are a measure of the extent of cellular elongation and hence discrimination of constricted pseudohyphae from parallel-sided hyphae, were determined as published (41). All measurements were made using the measurement tool in ImageJ 1.47v (http://imagej.nih.gov/ij), and MIs were calculated in Microsoft Excel.

### Calculation of the Surface Area (SA) of Fungal Cells

The SA of all cells was based on microscopical measurements made from DIC images using a Zeiss Axioplan 2 microscope and captured by a Hamamatsu C4742-95 digital camera (Hamamatsu Photonics, Hamamatsu, Japan). All measurements created by ImageJ were exported in Microsoft Excel. The SA of yeast cells was calculated based on measurements of the radius calculated from the average of the largest and smallest cell diameters of yeast cells (4π*r*^2^). Germ tube SA was taken as the SA of the mother yeast plus the SA of the daughter germ tube (true hypha). Hypha SA based on SA (2π*r*^2^ + 2π*rl*) where the germ tube length (*l*) was measured from the base of the mother cell and the germ tube diameter was the average of the narrowest and widest diameter measurements made along each germ tube. At least 50 measurements, and 3–4 biological replicates, of SA of individual yeast and hyphal cells grown were made.

### Cytokine Stimulation Assays

Blood samples were collected from healthy volunteers according to local guidelines and regulations, as approved by the College Ethics Review Board of the University of Aberdeen (CERB/2012/11/676). The PBMCs were used in this study and isolated using Ficoll-Paque™ PLUS (GE Healthcare) as previously described (44), with slight modifications. Unless otherwise indicated, 5 × 10^5^ PBMCs in 100 µl were incubated in a round-bottom 96-well plate (Nunc) with 100 µl of fungal cells at 1 × 10^6^ cells/ml. After incubation for 24 h at 37°C under 5% (v/v) CO_2_, plates were centrifuged at 1,000 *g* for 10 min at room temperature, and supernatants were saved and kept at −20°C until use. For cytokine assays using mixed *C. albicans* cell types, PBMCs were first preincubated with 50 µl of cells of one morphology at 2 × 10^6^ cells/ml for 1 h at 37°C under 5% (v/v) CO_2_. Subsequently, 50 µl of a sample containing a second sample of yeast or hyphae cells at 2 × 10^6^ cells/ml were added to PBMCs. Plates were then incubated for a further 24 h at 37°C, before supernatant collection and assay of induced cytokines. In experiments with PBMCs, the inoculum of hyphal cells was more or less aggregated. To assess the effect of cell aggregates on the cytokine stimulation, control experiments where hyphae were dispersed by ultrasonication before interaction with immune cells were performed, but theses did not show any significant differences with cultures of non-sonicated cells (data not shown). Therefore, steric blocking of monocyte access to fungal material did not explain the reduced response to hyphae that was observed.

TNFα, IL-1α, IL-1β, IL-6, and IL-10 concentrations were determined from coculture supernatants. For IL-1α quantification, stimulated PBMCs were disrupted by three sequential temperature shock cycles, and homogenates used for cytokine determination. All cytokine concentrations were determined using enzyme-linked immunosorbent assays (R&D Systems) according to the manufacturer’s instructions.

### Cell Wall Extraction and Analysis

*C. albicans* cells of different morphology were prepared as described above. Cells were collected by low-speed centrifugation and washed with ultrapure water, then broken using glass beads and a FastPrep machine (Qbiogene), homogenates were centrifuged at 13,000 *g* for 3 min, and pellets, containing the cell debris and walls, were washed five times with 1 M NaCl, resuspended in cell wall extraction buffer [50 mM Tris–HCl buffer pH 7.5, 2% (w/v) SDS, 0.3 M β-mercaptoethanol, and 1 mM EDTA], then boiled for 10 min, and washed three times with ultrapure water. Cell walls were freeze dried and stored at −20°C until used. The β-glucan, mannan, and chitin contents of cell wall preparations were determined by acid hydrolysis of the polymers and quantification of glucose, mannose, and glucosamine. Freeze-dried cell walls were hydrolyzed with 2 M trifluoroacetic acid as described previously (45), and acid hydrolyzates were analyzed by HPAEC-PAD (high-performance anion-exchange chromatography with pulsed amperometric detection) (46).

### hPBMC Cell Damage Assay

Human PBMC damage was assessed by lactate dehydrogenase (LDH) released into the supernatant in the culture medium. After 24 h stimulation with *C. albicans* either heat-killed yeast (HKY) or HKH over a range of MOIs (*Candida* cells:hPBMCs) from 0.002:1 to 2:1. The LDH release was determined using the cytotoxicity detection kit (Roche Applied Science), according to the manufacturer’s instructions. As a negative control for LDH release, 5 × 10^5^ cells of hPBMCs were incubated with only the cell culture medium and incubated at 37°C with 5% CO_2_ for 24 h. For the positive control maximum LDH release, 5 × 10^5^ cells of hPBMCs was obtained by treatment with 2% Triton X-100 and incubated under the same conditions. The percentage of LDH release was calculated relative to the value for 100% cell death.

### Statistical Analyses

The Mann–Whitney *U* test, *t*-test, or one-way ANOVA with a Dunnett’s *post hoc* test in an appropriate parameter was used to analyze data. Results are presented as means ± SDs or SEMs and levels of significance determined at *p* < 0.05.

## Results

### Differential Cytokine Induction by Yeast and Filamentous Cell Types

**Figure 1:**
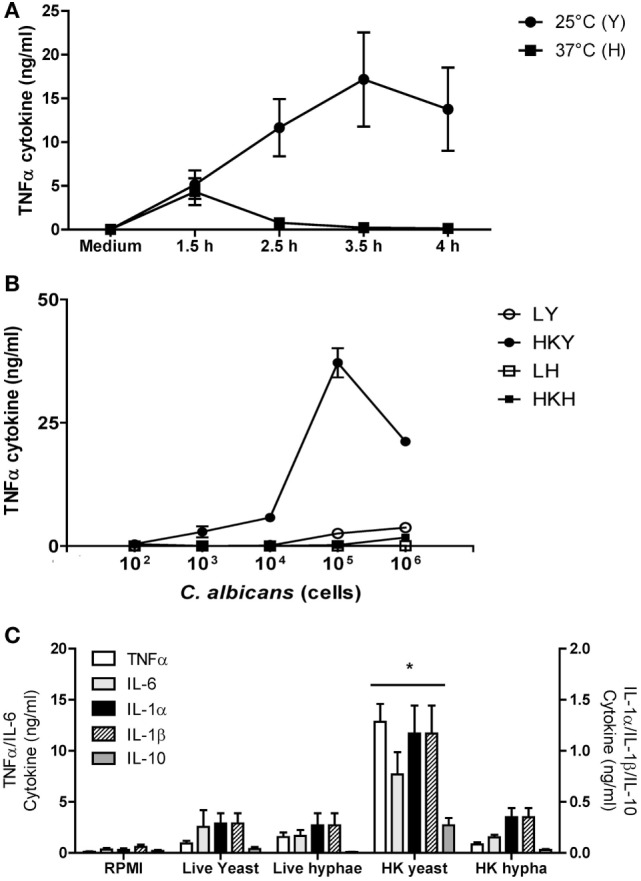
**TNFα stimulation with *Candida albicans* yeast and hyphal cells by human peripheral blood mononuclear cells (PBMCs)**. **(A)** Cytokine production by human PBMCs stimulated with yeasts cells (Y) of *C. albicans* NGY152 grown at 25°C (closed circles) or hyphae (H) grown at 37°C (closed squares) in RPMI 1640 + 2.5% fetal calf serum (FCS). Error bars = SEM (*n* = 3). **(B)** Dose-dependent stimulation of TNFα production by human PBMCs when stimulated with different forms of *C. albicans* NGY152 grown in RPMI 1640 + 2.5% FCS. Heat-killed yeasts (HKY), closed circles; live yeasts (LY), open circles; HK hyphae (HKH), closed squares; live hyphae (LH), open squares. Error bars = SEM (*n* = 6). In these experiments, a total of 5 × 10^5^ PBMCs were used in each treatment and so the MOI varied from 0.0002:1 to 2:1. **(C)** Cytokine production by human PBMCs stimulated with the different morphological forms of *C. albicans* NGY152. In each treatment, an inoculum of 1 × 10^6^ cells/ml was used. Results are means ± SEM (*n* = 6; *p < 0.05).

We used the cytokine response of human PBMCs as a read out to investigate the role of *C. albicans* morphogenesis on immune recognition. The cytokine profile of human PBMCs was compared when these immune cells were exposed to yeast cells and filamentous forms (true hyphae and pseudohyphae) of *C. albicans*. Yeast, pseudohyphal, and hyphal cells could all be induced *in vitro* using various culture conditions that preferentially stimulated a specific *C. albicans* cell morphology. Live and HK cells of different cell morphologies were also analyzed in our study. Across a wide range of conditions, heat killing increased the total amount of TNFα and other cytokines induced by yeast cells, and to a lesser extent, filamentous cells (Figure 1), suggesting that heat-treatment unmasks cytokine inducing PAMPs due to thermal disruption of components of the outer layer of the cell wall, thus exposing internal cytokine inductive cell layers to PRRs found on the surface of human PBMCs.

Under the conditions employed, more than 99% of yeast cells were obtained at 25°C and more than 94% of hyphae with at least two cell compartments were generated at 37°C (data not shown). Cell for cell, HK yeast cells induced significantly more TNFα than hyphal cells (Figure 1A). Germ tubes/hyphae induced less TNFα despite the presence of the parent yeast cell, implying that hypha formation may suppress TNFα production that would be normally associated with the cell surface of HK yeast cells (Figures 1A–C and 3).

TNFα production induced by *C. albicans* was dose dependent (Figure 1B). Live yeast cells stimulated lower cytokine production compared to HK yeast cells—a difference that was less apparent when comparing live and HK true hyphal cells (Figure 1B). Both live and HK hyphae stimulated poor TNFα production compared to that of HK yeast cells (Figures 1B,C). In addition, LDH activity released into the supernatant was measured after 24 h stimulation with *C. albicans* HKY or HKH. There was no significant difference between *C. albicans* stimulated and non-stimulated hPBMCs (Figure S1 in Supplementary Material). Therefore, although TNFα production by hPBMCs incubated with 1 × 10^6^ cells of HKY was significantly reduced, this was not due a loss of viability of hPBMCs. Similar results were obtained for a range of other cytokines, including IL-1α, IL-1β, IL-6, and IL-10 (Figure 1C).

**Figure 2:**
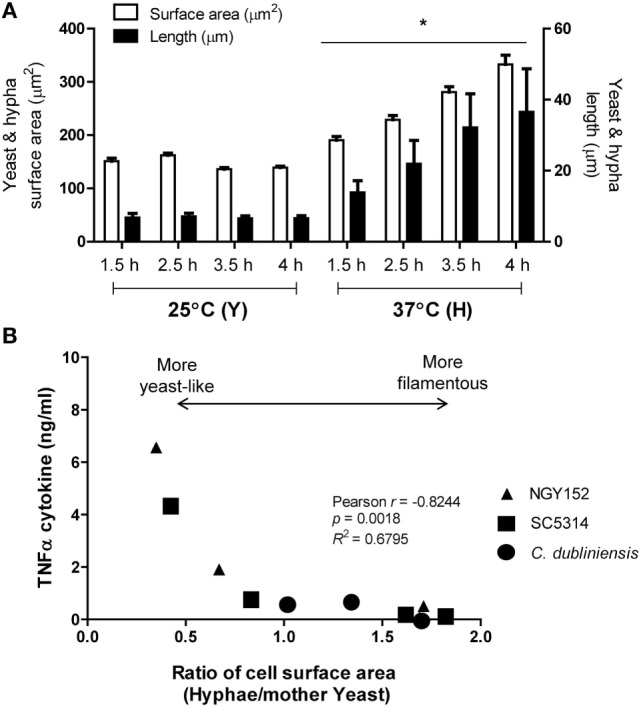
**Cell surface area (SA) of hyphal forming cells negatively correlated with TNFα stimulation**. **(A)** Cell SA and cell size of *Candida albicans* SC5314 yeast (Y) or hyphae (H) grown at either 25 or 37°C for 1.5, 2.5, 3.5, and 4 h (*p < 0.05). Error bars are SEM (*n* > 50 individual cells). **(B)** Relationship between TNFα production and extent of cell elongation expressed as the ratio of the hyphal cell area and the mother yeast cell area for *C. albicans* SC5314 and NGY152, and *Candida dubliniensis* CD36.

We next investigated how hypha cell SA correlated with TNFα production by human PBMCs. Cell SA and cell size (diameter or length) of *C. albicans* yeast and hyphae were calculated assuming yeast cells were elliptical spheres, and germ tubes were parallel-sided cylinders. The size and SA of yeast plus associated germ tubes cells grown at 25 and 37°C are shown in Figure 2A. SA and length of hyphae increased by time. Under these conditions (at 3.5 h) of growth, yeasts had a mean diameter of 6.6 ± 0.1 μm (mean ± SEM) and mean SA of 136 ± 3.1 μm^2^, respectively (Figure 2A). Hyphae had approximately twofold increased SA and were fivefold longer than the yeast cell diameter at the time cells were harvested. These values are comparable with those in previous reports (47). The average growth rate of hyphae grown in RPMI1640 plus 2.5% serum was 9.0 ± 1.1 μm/h. For *C. albicans* strains SC5314, NGY152, and the hypha-forming species *Candida dubliniensis* (strain CD36), there was a negative correlation between hypha to yeast cell surface ratio and TNFα production by human PBMCs (Figure 2B). Longer hyphae induced progressively less TNFα production per unit of cell surface (data not shown). We conclude that hyphae induce less TNFα than yeast cells and that the hypha surface may in some way suppress TNFα production by the cell wall of the attached parent yeast cell or that the yeast cell wall may be modified during the process of germ tube formation, so that it becomes less inductive of cytokine formation.

### Hypha Formation and Cytokine Induction by Human PBMCs

In order to confirm whether this observation was indeed related to cell morphology rather than the growth conditions used to generate yeast and hyphae, we compared the immune response of hyphae generated in different growth media. Live and HK hyphae generated in either YPD medium supplement with FCS, Lee’s medium, minimal medium (SC) added with GlcNAc, or in dilute FCS, were universally poorer inducers of TNFα, IL-1α, IL-1β, IL-6, and IL-10 (Table S1 in Supplementary Material) than yeast cells. Therefore, the reduced ability of *C. albicans* hyphae to stimulate cytokine production was independent of the hyphal-inducing growth conditions used.

**Figure 3:**
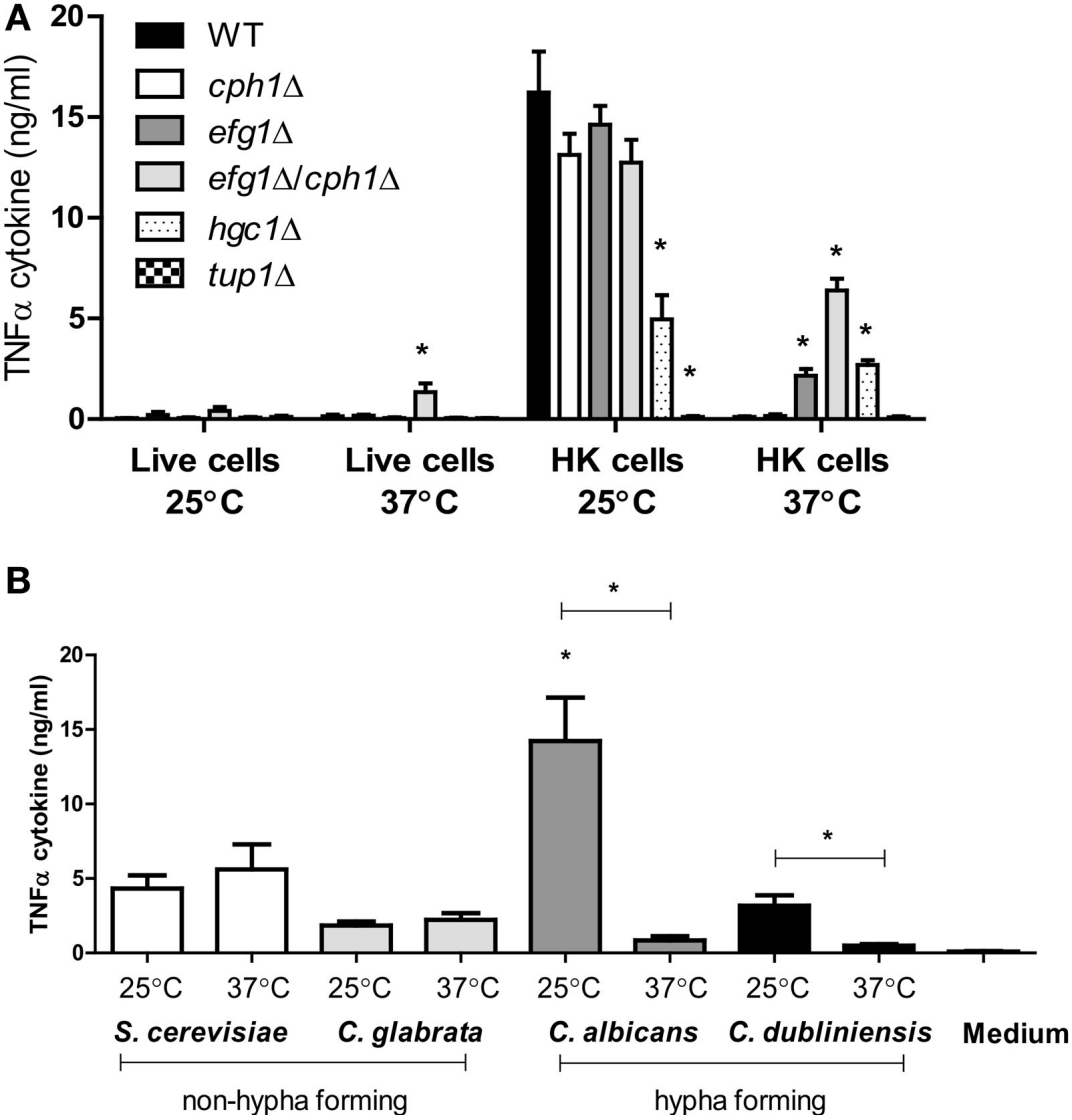
**TNFα production by human peripheral blood mononuclear cells stimulated with morphological mutants of *Candida albicans* mutants or other yeast species**. **(A)** *C. albicans* NGY152 (WT), JKC19 (*cph1*Δ), HLC52 (*efg1*Δ), HLC54 (*cph1*Δ/*efg1*Δ), WYZ12.2 (*hgc1*Δ), and Bca2-10 (*tup1*Δ). Results are means ± SEM (*n* = 6; *p < 0.05). **(B)** TNFα stimulation by non-hypha-forming species *Saccharomyces cerevisiae* (S288C) and *Candida glabrata* (ATCC 2001) compared to hypha-forming species *C. albicans* (NGY152) and *Candida dubliniensis* (CD36) (*p < 0.05). Error bars = SEM (*n* = 9).

Next, we investigated the TNFα simulation by yeast cells of *Candida glabrata* and *Saccharomyces cerevisiae* (48), which, unlike *C. albicans* and *C. dubliniensis*, are not able to form hyphae or pseudohyphae. Yeast cells of *S. cerevisiae, C. glabrata*, and *C. dubliniensis* stimulated comparable levels of TNFα cytokine when grown at 25 and 37°C (Figure 3B). *C. dubliniensis* yeast cells induced less TNFα than *C. albicans*, when grown at 25°C. However, as with *C. albicans, C. dubliniensis* yeast grown at 25°C induced more cytokine than hyphae grown at 37°C. Therefore, the hyphae of both *C. albicans* and *C. dubliniensis* stimulated less TNFα than the respective yeast form.

We then assessed the ability of various mutants that regulate morphogenesis to stimulate cytokine production. The *cph1*Δ mutant was still able to form hyphae under the experimental conditions used (93% hypha production), and these cells did not stimulate a high level of TNFα by human PBMCs (Figure 3A). In comparison, the *efg1*Δ mutant mainly produced pseudohyphae (more than 90%) and stimulated only about 15% of TNFα of the control HK wild-type yeast cells. The *cph1*Δ/*efg1*Δ double mutant was unable to form hyphae at 37°C and stimulated significantly higher amounts of TNFα at 37°C than the hyphal wild-type parent (Figure 3A). Another yeast-locked *hgc1*Δ mutant also induced higher levels of TNFα at 37°C, although surprisingly this was also reduced at 25°C, indicating that Hgc1 influences TNFα stimulation in human PBMCs for both yeast and hyphae. By contrast, the pseudohypha-locked *tup1*Δ mutant stimulated a poor cytokine production at both 25 and 37°C (Figure 3A). Similar results for TNFα were observed for IL-1α, IL-1β, IL-6, and IL-10 (data not shown).

**Figure 4:**
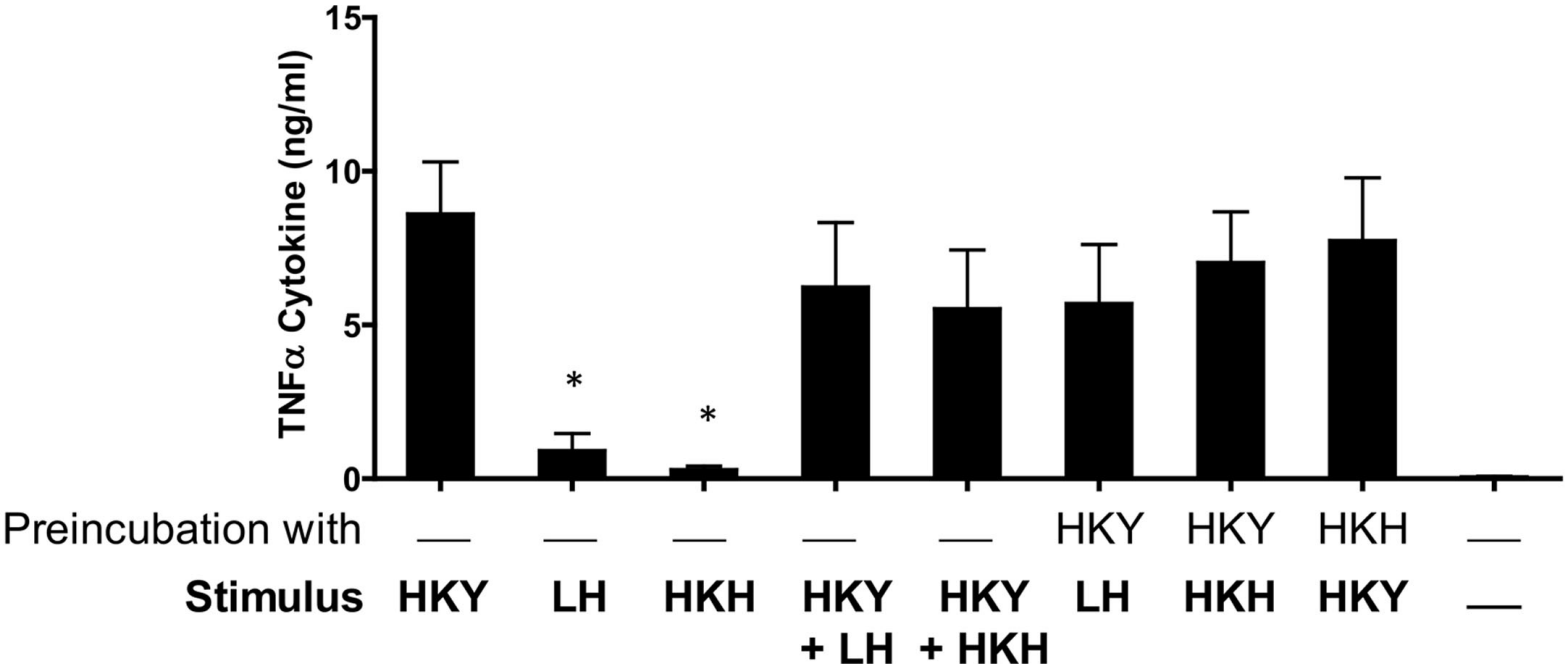
**Stimulation of cytokine production by human peripheral blood mononuclear cells (PBMCs) interacting with *Candida albicans* yeast cells and hyphae**. Human PBMCs in RPMI 1640 were preincubated 1 h with either *C. albicans* NGY152 yeast cells or hyphae and then stimulated with a second cell type for 24 h. HKY, heat-killed yeast; LH, live hyphae; HKH, heat-killed hyphae (*p < 0.05; *n* = 12).

Next, we investigated the apparent ability of the germ tube to inhibit the ability of the mother yeast cells to stimulate TNFα cytokine production. PBMCs were pretreated with live or HK yeast or hyphae for 1 h, then a second stimulus of the same or another morphotype was added. PBMCs preincubated with HK yeast cells and then stimulated with either live or HK hyphae were not compromised in their ability to produce TNFα (Figure 4). Reciprocally when HK hyphae were preincubated with PBMCs and then stimulated with HK yeast cells, there was a no significant reduction in the cytokine response (Figure 4). When yeast cells and hyphae were mixed together in a ratio 1:1, there was a strong cytokine production comparable to that elicited with HK yeast cells alone. Therefore, germ tubes with an attached parent yeast cell did not induce a response that would normally be associated with free yeast cells; however, hyphae did not block cytokine stimulation by yeast cells presented in trans.

The possibility that *C. albicans* reduced cytokine production by hyphae was due to blocking receptors on immune cells was tested by coincubating hyphal cells with various TLR ligands including Pam_3_CSk_4_, LPS, zymosan, flagellin, and curdlan. However, *C. albicans* hyphae did not block nor reduce TNFα, IL-1β, or IL-1α stimulated by these TLRs ligands used (Figure S2 in Supplementary Material).

### Cell Wall Composition and the Immune Response

**Figure 5:**
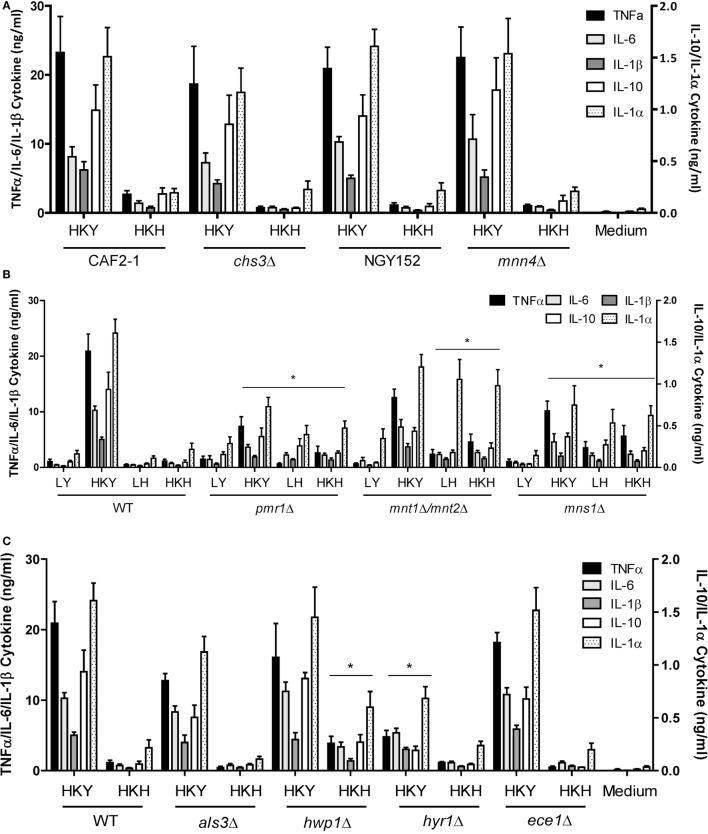
**Cytokine production stimulated by cell wall mutants of yeast and hyphal cells**. **(A)** Cytokine production stimulated by human PBMCs with LY—live yeast; HKY—heat-killed yeast; LH—live hyphae; HKH—heat-killed hyphae; *Candida albicans mnn4*Δ and *chs3*Δ and control strains, NGY152 and CAF2-1. **(B)** Cytokine stimulation with mannosylation defective mutants, *pmr1*Δ, *mnt1*Δ/*mnt2*Δ, and *mns1*Δ. **(C)** *als3*Δ, *hwp1*Δ, *hyr1*Δ, and *ece1*Δ. Results presented as mean ± SEM (*n* = 6), and the asterisks indicate comparison of the wild-type values and the mutant strains (*p < 0.05).

The fungal cell wall contains most of the PAMPs recognized by the innate immune cells (4, 11). Thus, we next assessed the ability of yeast and hyphal cells with specific cell wall defects to stimulate cytokine production by human PBMCs. A *chs3*Δ null mutant with a low chitin content at the cell wall (49), and a *mnn4*Δ mutant (50) lacking cell wall mannosylphosphate, were unaffected in the PBMC-induced cytokine production, compared to wild-type control cells (Figure 5A). A *pmr1*Δ mutant (51), which is deficient in yeast and hyphal cell wall *N*- and *O*-linked mannan, induced a reduced cytokine response by HK yeast cells, but an increased cytokines response from live and HK hyphae. This suggests that for yeast cells the lack of mannan reduced the overall immune response, while in hyphae the major effect of mannan depletion was to reveal subsurface immunostimulatory ligands such as β-glucan. In the *O*-mannosylation *mnt1/mnt2*Δ mutant (52), and the core *N*-mannan *mns1*Δ mutant (45) the secretion of most cytokines was enhanced or not affected in yeast or hyphal cells. IL-1α secretion from monocytes was enhanced to the greatest extend in the *pmr1*Δ, *mnt1/mnt2*Δ, and *mns1*Δ mutants in both yeast and hyphal cells (Figure 5B).

We compared the cytokine levels produced by a number of mutants that lacked hypha-associated cell wall proteins including *als3*Δ (53), *hwp1*Δ (54), *hyr1*Δ (55), and *ece1*Δ (56) with those stimulated with wild-type control cells. Only the *hwp1*Δ HK mutant hyphae stimulated a higher cytokine production than wild-type control cells (Figure 5C). Unexpectedly, despite the hypha-specific expression pattern for *HYR1* (55), the *hyr1*Δ mutant yeast cells stimulated a slightly reduced cytokine response from human PBMCs compared to wild-type control cells (Figure 5C). The *hwp1*Δ mutant showed no significant differences in mannan, glucan, and chitin content of the cell wall, when compared to wild-type control cells in both morphologies (Table 1), suggesting that this proteins may act in masking cytokine stimulating PAMPs in the cell wall. By contrast, yeast and hyphal cells of the *mns1*Δ and *pmr1*Δ mutants displayed a significant reduction in cell wall mannan and increased levels of glucan (Table 1). Therefore, cell wall mannosylation and the presence of Hwp1 were important for the reduced ability of hyphae of *C. albicans* to induce cytokines by human PBMCs.

**Table 1:** **Cell wall composition of *Candida albicans* strains**.

Strain	Glucosamine	Glucose	Mannose
**Yeast morphology**			
WT	1.01 ± 0.4	58.31 ± 2.25	40.68 ± 2.19
*hwp1*Δ	0.77 ± 0.46	52.99 ± 0.77*	46.24 ± 0.34*
*mns1*Δ	0.91 ± 1.18	88.71 ± 2.34*	10.38 ± 0.96*
*pmr1*Δ	1.50 ± 2.05	99.55 ± 3.63*	0.50 ± 1.33*
**Hypha morphology**			
WT	4.77 ± 0.32^#^	87.18 ± 1.54^#^	8.06 ± 0.99^#^
*hwp1*Δ	5.63 ± 0.93^#^	83.68 ± 2.57^#^	10.68 ± 1.75^#^
*mns1*Δ	6.10 ± 1.34^#^	87.61 ± 1.59	6.29 ± 0.18^#^
*pmr1*Δ	1.48 ± 0.74*	97.72 ± 2.01*	0.80 ± 0.68^*,#^

Means ± SD (*n* = 3) *p < 0.05 when comparing the mutant strain with the wild-type control cells.

*^#^p < 0.05 when comparing hyphae with yeast cells*.

We also tested the effect of deletion of the yeast-specific gene *PGA29* on cytokine production by human PBMCs and noted that this HK mutant induced less TNFα under conditions of yeast growth (Figure S3 in Supplementary Material). The reconstituted heterozygous *pga29/PGA29* mutant restored the TNFα induction to normal levels. Therefore, morphology-specific cell wall proteins of both yeast and hyphae influenced immune recognition.

### *C. albicans* Pseudohyphae Stimulate Intermediate Cytokine Levels from Human PBMCs

**Figure 6:**
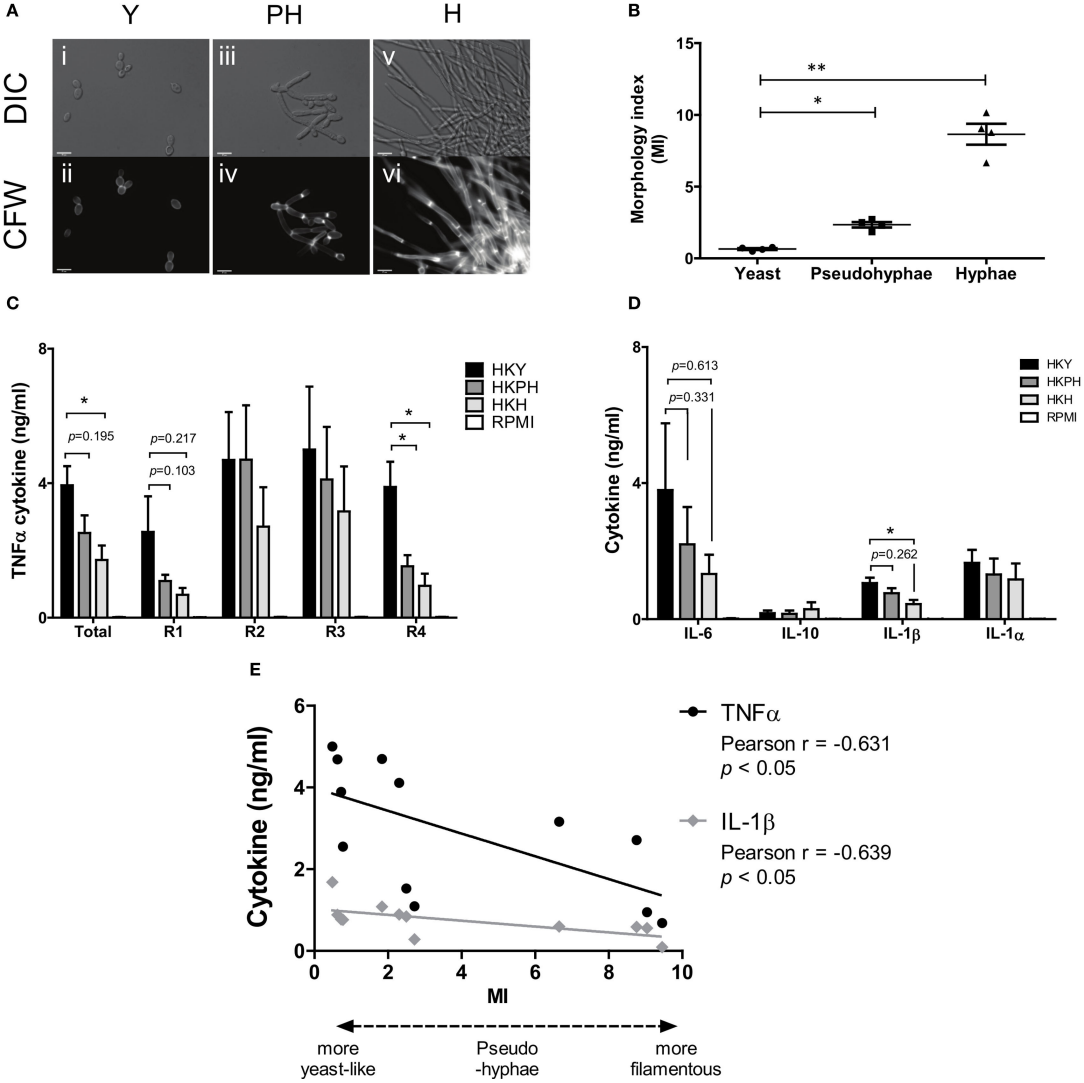
**Cytokine production by hPBMCs stimulated by different *Candida albicans* morphological forms**. **(A)** Yeasts (i–ii), pseudohyphae (iii–iv), or hyphae (v–vi) of *C. albicans* NGY152 were grown in RPMI 1640 (pH 7) for 6 h at 25, 30, and 37°C, respectively, fixed, and stained with 25 µg/ml CFW to visualize the cell wall. Scale bars = 10 µm. **(B)** Morphological index (MI) of yeast, pseudohyphal, and hyphal cells was measured. Error bars are SEMs (averages from 50 cell measurements and 4 biological replicates) (*p < 0.05; **p < 0.01). **(C)** TNFα cytokine production elicited by human peripheral blood mononuclear cells (PBMCs) with HK NGY152 of yeast (Y), pseudohypha (PH), and hypha (H) cells. Each replicate (R1–R4) were averaged as total values. Data are means ± SEM (*n* > 6; *p < 0.05). **(D)** IL-6, IL-1β, IL-1α, and IL-10 cytokine production elicited by human PBMCs with HK of yeast (Y), pseudohypha (PH), and hypha (H) cells. Data are means ± SEM (*n* > 6; *p < 0.05). **(E)** Correlation between TNFα or IL-1β cytokine and MI. Results are Person *R* values (*n* = 4 biological replica).

We then examined the ability of pseudohyphae to induce cytokine production. We deployed a method in which changes in temperature alone could generate yeast cells, pseudohyphae, or hyphae (41, 57). When *C. albicans* NGY152 was grown in RPMI 1640 medium at a neutral pH and at 30°C for 6 h (Figure 6A), it reproducibly resulted in a largely pseudohyphal population (~90%). For this strain, growth at 25°C yielded yeast cells (MI = 0.65 ± 0.06, mean ± SEM), 30°C yielded pseudohyphae (MI = 2.34 ± 0.19), and 37°C yielded hyphal forms (MI = 8.65 ± 0.73) (Figure 6B).

Next, we investigated cytokine production of human PBMCs stimulated with pseudohyphae in comparison to yeast and hyphae. In a range of experiments pseudohyphal cell populations generated reproducibly intermediate levels of TNFα and other cytokines from live and HK cells, although the differences were not always significant at *p* < 0.05 and the level of significance varied depending on whether the average MI of a given population of pseudohyphae was sufficiently distinct from that of populations of yeast cells or hyphae (Figures 6A–D). There was a statistically significant correlation between the level of TNFα and IL-1β cytokine from PBMCs and the MI of *C. alb*icans cells inducing these cytokines (Figure 6E).

## Discussion

Multiple independent studies have proposed a positive correlation between the formation of *C. albicans* hyphae and an enhanced capacity for tissue invasion, damage, and virulence (6, 33, 39). However, the significance of *C. albicans* morphogenesis on the innate immune response has not been fully characterized. Here, the interaction of yeasts, pseudohyphae, and hyphae of this fungus with cells of the human innate immune system was investigated using cytokine production by human PBMCs as an immunoassay readout. We demonstrate that yeast cells generated more inflammatory cytokines from PBMCs than hyphae, and that pseudohyphae generated intermediate cytokine levels. These differences were observed in independent strains for cells generated in different growth media. Heat killing of cells has been used frequently in immunological studies of *C. albicans* to prevent cells undergoing filamentation in response to serum components of cell culture media when exposing *Candida* to immune cells during cytokine induction assays (58, 59). HK cells had enhanced immune responses, which has been interpreted as being due to heat-induced permeabilization of the cell wall and subsequent exposure of the underlying β1,3-glucan layer, which is strongly immunogenic (18). Our data support this hypothesis and suggest that HK cells generate a greater cytokine signal because more PAMPs can engage collaboratively with PRRs, thus resulting in coreceptor amplification of the cytokine response (18, 60–62).

Although the hyphae used in these experiments also had a parental yeast cell, the combined cytokine signal due to the hypha plus parent yeast cell was significantly less than that expected from the yeast cell alone. This may suggest that either the yeast cell surface of germ tube matures to become different from that of a free yeast cell or that an unknown mechanism operates in germ tubes that are able to block cytokine induction due to the mother yeast cell. However, if a blocking signal is present, it does not operate in “trans” since hyphae added after free yeast cells were used to stimulate PBMCs did not interfere with the ensuing yeast cell stimulated cytokine response. Interestingly, hyphae of *C. dubliniensis* also stimulated less cytokine from PBMCs than yeast cells. The genomes of *C. albicans* and *C. dubliniensis* are 95% identical, and the cell walls are also thought to be of similar composition, although there are notable differences in the cell wall proteome (63). These closely related *Candida* species therefore show both common aspects and some differences in the nature of the immune response to yeast and hyphal cells.

Different immune cell types respond differently to *C. albicans* yeast and hyphae. For example, *C. albicans* hyphae induce higher levels of TNFα than yeast cells in macrophages (64, 65), while yeast and hyphae stimulate comparable levels of IL-8 cytokine by human neutrophils (47). It was shown that *C. albicans* induced different cytokine responses from oral and vaginal epithelial cells, and that hyphae induced higher cytokine levels than yeast cells in both epithelial cell types (32, 66). However, our findings reinforce previous studies where *C. albicans* filaments (hyphae and pseudohyphae) were reported to stimulate the production of less IL-12, IFN-γ, IL-1β, and IL-12p70 by human PBMCs and murine splenic lymphocytes than *C. albicans* yeast cells (30, 58, 67–69). It was also previously reported that TLR4-mediated pro-inflammatory signals were diminished during the germination of *C. albicans* yeast cells into hyphae (30). Supporting this, we show that *C. albicans* cells grown at 37°C have a progressively reduced TNFα cytokine response. In addition, it was reported that *C. albicans* hyphal cell walls also stimulated less chemokines than yeast cell walls (68), and it was suggested that this may be due to surface expression of β1,6-glucan being lower on hyphae compared to yeast cells. The cell SA of each individual hyphal compartment is larger than that of a yeast cell, and hyphae have no bud scars where inner wall layers are exposed (29). Therefore, it is possible that the density of certain immune agonists is less concentrated on the hyphal surface than on yeast cells. Also, progressive elongation of hyphae *in vivo* has been shown to result in increasing exposures of β1,3-glucan (70). Hence, cellular morphogenesis leading to filamentous growth of *C. albicans* leads to important progressive modifications of cell wall composition and architecture that has profound and differing effects on the immune response.

Cell wall polysaccharide analysis showed that yeast cell walls contained significantly higher amounts of mannan but lower amounts chitin and glucan compared to that of hyphal cell walls (Table 1). The reduction of mannan and increase in chitin content of hyphal cell walls might also be related to the lower cytokine responses to *C. albicans* hyphae (28, 45, 71, 72). Our results underline the importance of *N*- and *O*-linked mannans in the recognition of *C. albicans* hyphae by human PBMCs (Figure 5) since *N*- and *O*-mannan mutants (45, 52), but not chitin and phosphomannan mutants, stimulated the production of higher levels of cytokines that wild-type hyphae.

Although the primary polysaccharides in the cell wall are likely to have a major influence on immune recognition, it was noted that a HK *hwp1*Δ cell wall protein deletion mutant induced an increased cytokine signal, despite having no measurable alteration in hyphal mannan or glucan content. Similarly, a mutant lacking *MNS1* grown under hyphal-inducing conditions showed insignificant changes in cell wall components, but substantial reduction in cytokine production. However, the *mns1* yeast cells had 34% increased glucan as a compensation of 70% reduction in mannan, and a reduced cytokine profile. Therefore, there was no direct or universal correlation between cytokine induction and gross cell wall polysaccharide composition.

Our observations suggest that surface proteins, polysaccharides, and virulence factors are regulated or modified during filamentous growth resulting in changes in the immune response. Such changes in the incorporation of surface cell wall proteins on cells of different morphology could mask or unmask PAMPs, thereby blocking or promoting PRR engagement.

The presence of *C. albicans* hyphae could potentially compete with yeast cells for the ability to bind PRRs and stimulate immune cells. This would be important if specific yeast cell wall proteins are important for immune recognition and activation. We observed that a *pga29* mutant grown in the yeast form stimulated less TNFα from human PBMCs. This cell wall protein Pga29 has homologs in several pathogenic *Candida* spp. and is abundant in yeast cell walls in *C. albicans* but not in hyphae (73). Deletion of *PGA29* resulted in decreased glucan–mannan in the cell wall, and reduction of TNFα, IL-6, and IL-8 stimulated by oral reconstituted human epithelial cells (74). Therefore, both yeast and hypha-specific cell wall proteins may directly modulate immune responses.

It is also possible that *C. albicans* hyphal cells may produce secreted molecules that suppress immune recognition. Quorum-sensing molecules, such as farnesol, tyrosol, phenylethanol, and tryptophol, produced by *C. albicans*, play a key role in morphogenesis (75–77), and tyrosol acts negatively on cytokine production stimulated by RAW 264.7 macrophages induced by lipopolysaccharide (78). However, we showed that neither live nor HK hyphae could suppress yeast cell-induced cytokine production in trans; therefore, soluble factors were not suggested as playing a significant role in our experiments.

Our observations and those of previous studies (30, 58, 68, 69) demonstrate that *C. albicans* hyphae stimulated lower cytokine production by PBMCs than *C. albicans* yeast cells, thus indicating that *C. albicans* hyphae may help to evade or alter the host immune response. However, it is clear that *C. albicans* hyphae induced strong cytokine responses and caused more damage to epithelial cells, while yeast cells did not trigger cytokine responses (66, 79, 80). Also, *C. albicans* hyphae induce stronger cytokine responses that yeast cells from macrophages (64, 65). The common denominator of these various reports is that cellular morphogenesis plays an important role in determining the immune response to *C. albicans*, but the nature of this response is both host cell type specific and pathogen morphotype dependent. These data underline the perspective that *C. albicans* presents a moving target to the cells of the innate immune response.

## Ethics Statement

Blood samples were used in this study to generate human peripheral blood monocytes. Samples were collected from healthy volunteers according to local guidelines and regulations, as approved by the College Ethics Review Board of the University of Aberdeen (CERB/2012/11/676).

## Author Contributions

LM and KL performed experiments. NG, HM-M, LM, and KL conceived and designed experiments and analyzed the data. NG and HM-M supervised the project. LM, KL, and NG contributed to writing of the manuscript. All the authors reviewed the manuscript.

## Conflict of Interest Statement

The authors declare that the research was conducted in the absence of any commercial or financial relationships that could be construed as a potential conflict of interest.

## Supplementary Material

Figure S1 **Cell damage assay of hPBMC stimulated with *Candida albicans***. Lactate dehydrogenase activity released from 5 × 10^5^ hPBMCs into the culture medium was determined after 24 h stimulated with either *C. albicans* heat-killed yeast or HKH at the different number of cells from 1 × 10^2^ to 1 × 10^6^ cells. The result represented as a percentage relative to 100% cell death of hPBMCs when killed with 2% Triton-X-100. Error bars = SEM (*n* = 4).

Figure S2 **Cytokine production by hPBMCs stimulated by different *Candida albicans* coincubated with toll-like receptors (TLRs) ligands**. Human peripheral blood mononuclear cells were stimulated using a mixture of *C. albicans* cells and TLRs ligands. Cells were either live (L) or heat-killed (HK), and yeast (Y) or hyphae (H). TLRs ligands used were flagellin (for TLR5), LPS (for TLR4), Pam_3_CSK_4_ (for TLR2/TLR1), zymosan, and curdlan (for Dectin-1). The cytokines measured were TNF-α **(A)**, IL-6 **(B)**, IL-1β **(C)**, IL-1α **(D)**, and IL-10 **(E)**. IL-10 was barely detectable and could not be evaluated. Data are means ± SEM (*n* > 3; **p* < 0.05). ND, not detectable.

Figure S3 **TNFα cytokine production by hPBMCs incubated with *Candida albicans* lacking *PGA29***. *C. albicans* cells lacking *PGA29* was grown in RPMI 1640 + 2.5% fetal calf serum at 25 or 37°C for 3.5 h. Cells were collected and heat killed. The mutant was incubated with hPBMCs for 24 h. Then, TNFα cytokine production was determined (see Methods and Materials). Data are means ± SEM (*n* > 9; **p* < 0.05; ****p* < 0.001).

Table S1 ***C. albicans* and other fungal strains used in this study**.
