# Supplementary figures and images for: Candida albicans Yeast, Pseudohyphal, and Hyphal Morphogenesis Differentially Affects Immune Recognition
Source: Front Immunol. 2017 Jun 7;8:629. doi: 10.3389/fimmu.2017.00629 (PMC5461353; doi:10.3389/fimmu.2017.00629)

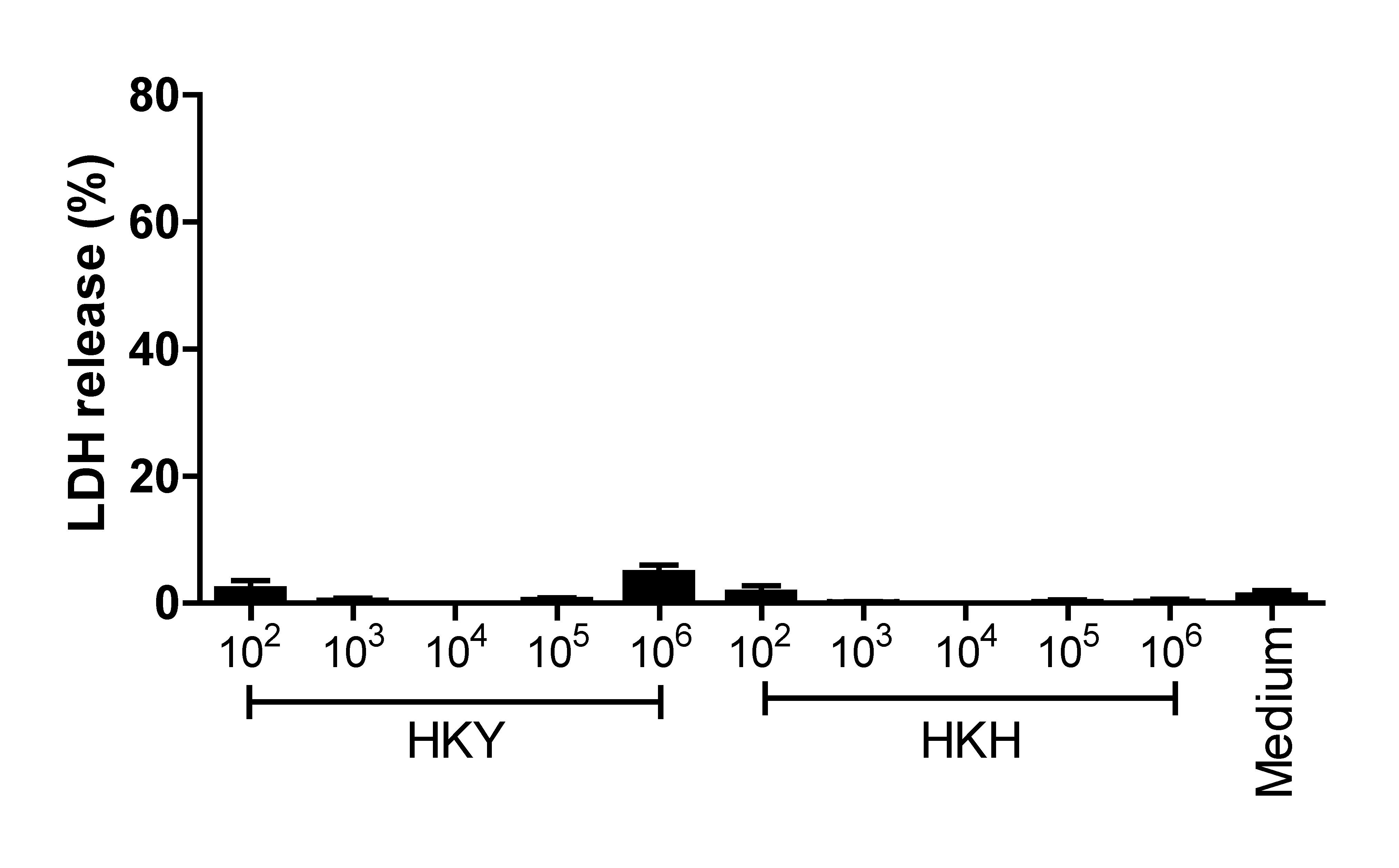

Supplement: Figure S1 — Cell damage assay of hPBMC stimulated with Candida albicans. Lactate dehydrogenase activity released from 5 × 10^5^ hPBMCs into the culture medium was determined after 24 h stimulated with either C. albicans heat-killed yeast or HKH at the different number of cells from 1 × 10^2^ to 1 × 10^6^ cells. The result represented as a percentage relative to 100% cell death of hPBMCs when killed with 2% Triton-X-100. Error bars = SEM (n = 4). [file Image_1.TIF]

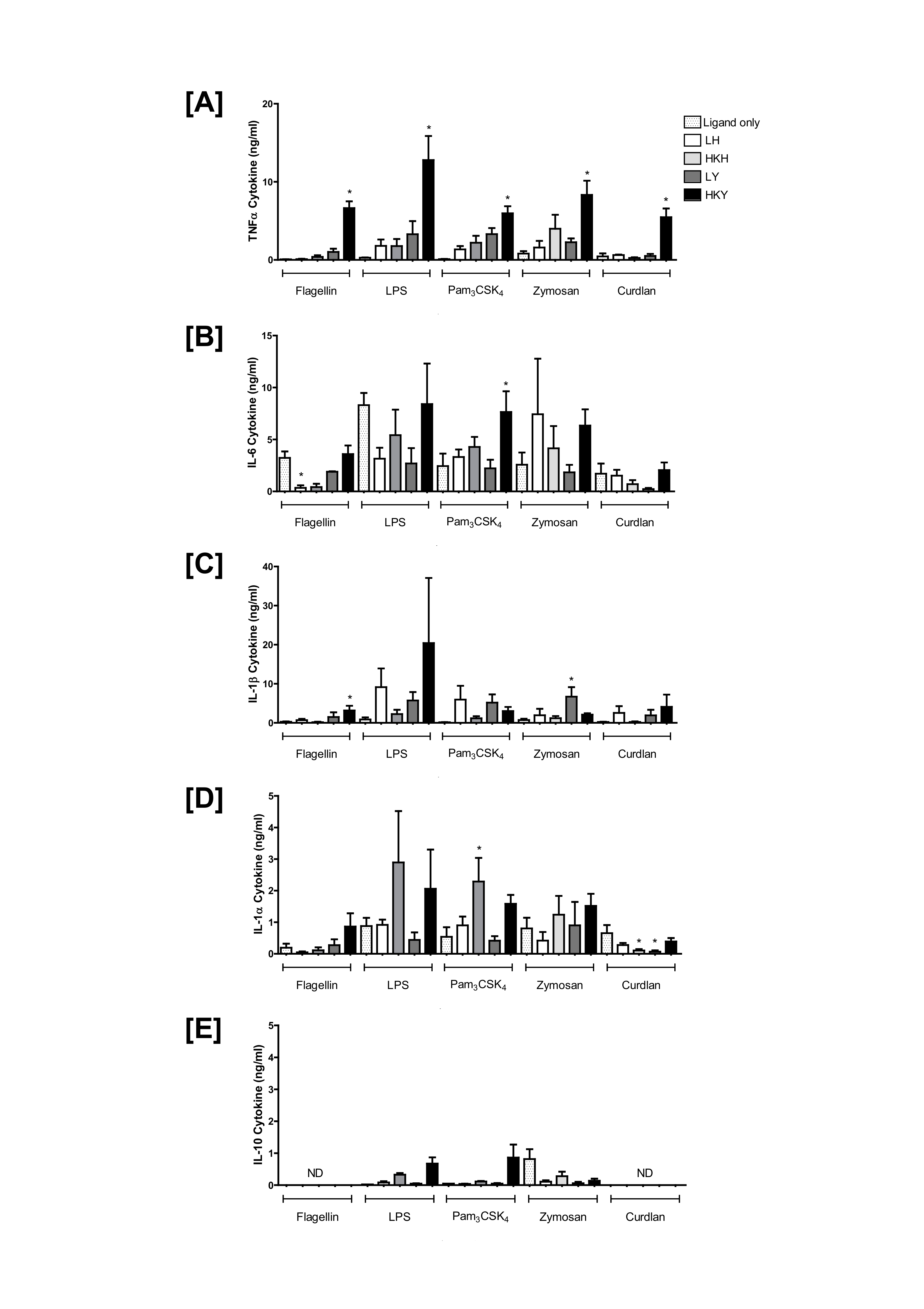

Supplement: Figure S2 — Cytokine production by hPBMCs stimulated by different Candida albicans coincubated with toll-like receptors (TLRs) ligands. Human peripheral blood mononuclear cells were stimulated using a mixture of C. albicans cells and TLRs ligands. Cells were either live (L) or heat-killed (HK), and yeast (Y) or hyphae (H). TLRs ligands used were flagellin (for TLR5), LPS (for TLR4), Pam3CSK4 (for TLR2/TLR1), zymosan, and curdlan (for Dectin-1). The cytokines measured were TNF-α (A), IL-6 (B), IL-1β (C), IL-1α (D), and IL-10 (E). IL-10 was barely detectable and could not be evaluated. Data are means ± SEM (n > 3; *p < 0.05). ND, not detectable. [file Image_2.TIF]

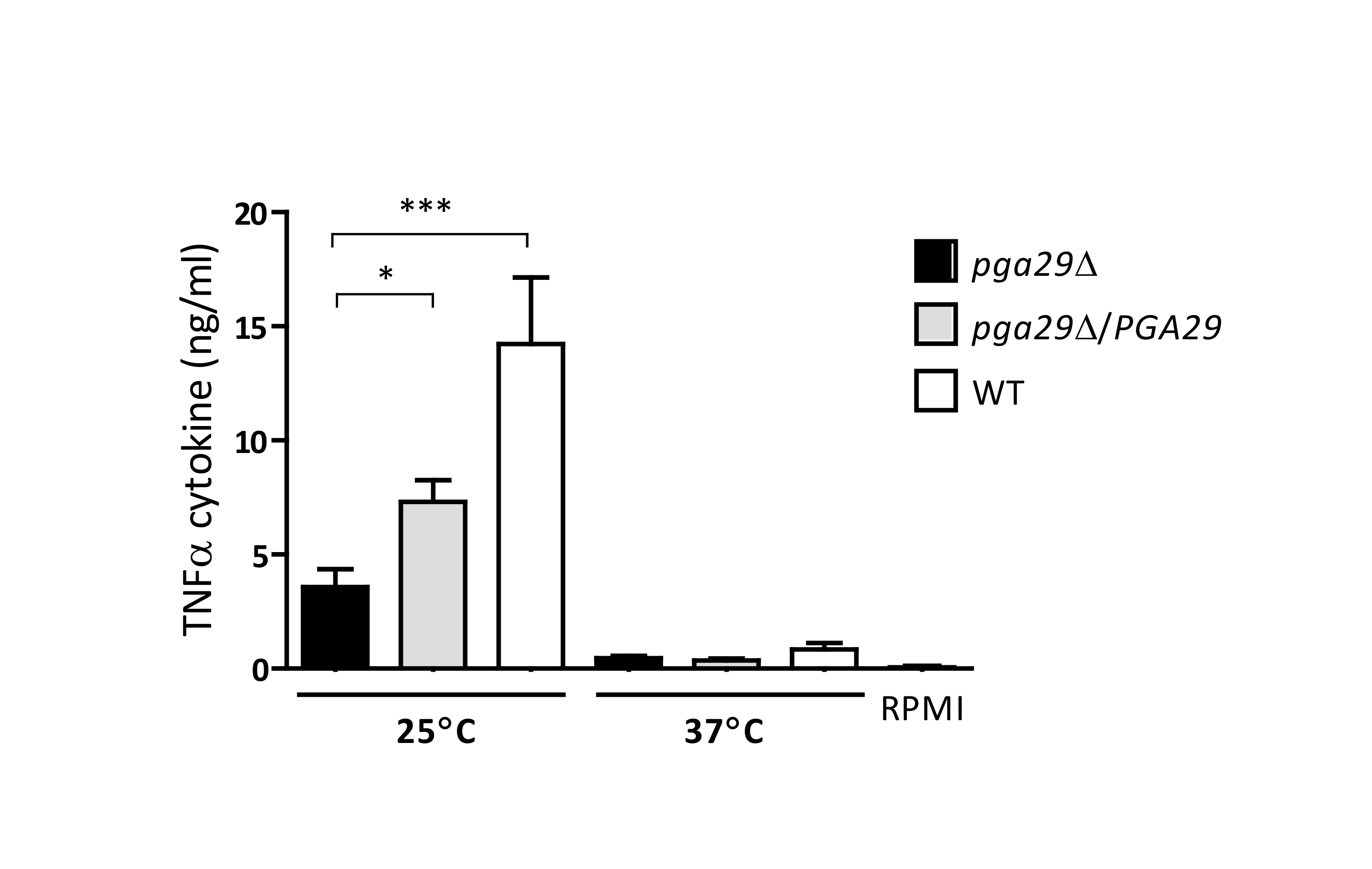

Supplement: Figure S3 — TNFα cytokine production by hPBMCs incubated with Candida albicans lacking PGA29. C. albicans cells lacking PGA29 was grown in RPMI 1640 + 2.5% fetal calf serum at 25 or 37°C for 3.5 h. Cells were collected and heat killed. The mutant was incubated with hPBMCs for 24 h. Then, TNFα cytokine production was determined (see Methods and Materials). Data are means ± SEM (n > 9; *p < 0.05; ***p < 0.001). [file Image_3.TIF]
