# Supplementary material for: Candida albicans Yeast, Pseudohyphal, and Hyphal Morphogenesis Differentially Affects Immune Recognition
Source: Front Immunol. 2017 Jun 7;8:629. doi: 10.3389/fimmu.2017.00629 (PMC5461353; doi:10.3389/fimmu.2017.00629)
Supplement: Table S1 — C. albicans and other fungal strains used in this study. [file Data_Sheet_1.doc]

Suppl. Table 1. *C. albicans* and other fungal strains used in this study

| Designation  (Published Name) | Genotype | Reference |
| --- | --- | --- |
| *C. glabrata*  CBS138 | Clinical isolate | CBS* |
| *C. dubliniensis*  CD36 | Clinical isolate | (1) |
| *S. cerevisiae*  BY4741/S288C | MATa *his3*Δ*1* *leu2*Δ*0* *met15*Δ*0* *ura3*Δ*0* | (2) |
| UC820 | Clinical isolate | (3) |
| AM2005/0387 | Clinical isolate | (4) |
| SCS 503505 | Clinical isolate | (4) |
| SC5314 | Clinical isolate | (5) |
| Wild type  (NGY152) | *ura3*Δ::*imm434/ura3*Δ::*imm434 RPS1/rps1*::*URA3* | (6) |
| Wild type  (parent of *chs3*Δ)  (CAF2-1) | *ura3*Δ::*imm434/URA3* | (7) |
| *efg1*Δ  (HLC52) | *ura3*Δ*::imm434/ura3*Δ*::imm434 efg1*Δ*::hisG/efg1*Δ*::hisG-URA3-hisG* | (8) |
| *cph1*Δ  (JKC19) | *ura3*Δ*::imm434/ura3*Δ*::imm434 cph1*Δ*::hisG/cph1*Δ*::hisG-URA3-hisG URA3* | (9) |
| *chp1*Δ/*efg1*Δ  (HLC54) | *ura3*Δ*::imm434/ura3*Δ*::imm434 cph1*Δ*::hisG/cph1*Δ*::hisG efg1*Δ*::hisG/efg1*Δ*::hisG-URA3-hisG* | (8) |
| *hgc1*Δ  (WYZ12.2) | *ura3*Δ*::imm434/ura3*Δ*::imm434 hgc1*∆::*ARG4*/*hgc1*∆::*HIS1*, URA3 | (10) |
| *ece1*Δ  (CAF6-8) | *ura3*Δ*::imm434/ura3*Δ*::imm434 ece1*Δ*::hisG(I-SceI)-URA3-hisG(I-SceI)/ece1*Δ*::hisG(I-SceI)* | (7) |
| *pmr1*Δ  (NGY355) | *ura3*∆::*imm434/ura3*∆::*imm434 pmr1*∆::*hisG/pmr1*∆::*hisG RPS1/rps1*∆::CIp10 | (11) |
| *mnn4*Δ  (CHD15) | *ura3*∆::*imm434/ura3*∆::*imm434 mnn4*∆::*hisG/mnn4*∆::*hisG RPS1/rps1*∆::CIp10 | (12) |
| *mns1*Δ  (HMY5) | *ura3*∆::*imm434/ura3*∆::*imm434 mns1*Δ*::dp1200/mns1*Δ*::dp1200 RPS1/rps1*Δ*::*CIp10 | (13) |
| *mnt1-mnt2*Δ  (NGY337) | *ura3*∆::*imm434/ura3*∆::*imm434*  *mnt1-mnt2*Δ*::hisG/mnt1-mnt2*Δ*::hisG RPS1/rps1*Δ*::*CIp10 | (14) |
| *chs3*Δ  (myco3) | *ura3*Δ*::λimm434/ura3Δ::λimm434 chs3*Δ*::hisG/ch3*Δ*::hisG-URA3-hisG* | (15) |
| *hwp1*Δ  (CAH7-1A1E2) | *ura3*Δ::*imm434/ura3*Δ::*imm434 hwp1*Δ::*hisG/hwp1*Δ::*hisG eno1*::*URA3* | (16) |
| *hyr1*Δ | *ura3*Δ::*imm434/ura3*Δ::*imm434 hyr1*Δ::*hisG*/Δ*hyr1Δ*::*hisG* | (17) |
| *als3*Δ  (1843) | *ura3*Δ::*imm434/ura3*Δ::*imm434 als3la*Δ/*als3sa*Δ-*URA3* | (18) |
| *pga29Δ* | *pga29*Δ*/pga29*Δ*, pga29::HIS1/pga29::ARG4,*  *rps1::URA3* | (19) |
| *pga29Δ*/*PGA29* | *pga29*Δ*/pga29*Δ*, pga29::HIS1/pga29::ARG4,*  *rps1::URA3* | (19) |
| * CBS: <http://www.cbs.knaw.nl/Collections/> | | |

**References**

1. Sullivan DJ, Westerneng TJ, Haynes KA, Bennett DE, Coleman DC. *Candida dubliniensis* sp. nov.: phenotypic and molecular characterization of a novel species associated with oral candidosis in HIV-infected individuals. *Microbiology* (1995) 141:1507–21. doi: 10.1099/13500872-141-7-1507 PMID:7551019

2. Brachmann CB, Davies A, Cost GJ, Caputo E, Li J, Hieter P, et al. Designer deletion strains derived from *Saccharomyces cerevisiae* S288C: a useful set of strains and plasmids for PCR-mediated gene disruption and other applications. *Yeast* (1998) 14:115–32. doi: 10.1002/(SICI)1097-0061(19980130)14:2<115:AID-YEA204>3.0.CO;2-2 PMID:9483801

3. Cheng SC, van de Veerdonk FL, Lenardon M, Stoffels M, Plantinga T, Smeekens S, et al. The dectin-1/inflammasome pathway is responsible for the induction of protective T-helper 17 responses that discriminate between yeasts and hyphae of *Candida albicans*. *J Leukoc Biol* (2011) 90:357–66. doi: 10.1189/jlb.1210702 PMID:NOPMID

4. Netea MG, Gow NAR, Joosten LA, Verschueren I, van der Meer JW, Kullberg BJ. Variable recognition of *Candida albicans* strains by TLR4 and lectin recognition receptors. *Med Mycol* (2010) 48:897–903. doi: 10.3109/13693781003621575 PMID:20166865

5. Gillum AM, Tsay EY, Kirsch DR. Isolation of the *Candida albicans* gene for orotidine-5'-phosphate decarboxylase by complementation of *S. cerevisiae* *ura3* and *E. coli pyrF* mutations. *Mol Gen Genet* (1984) 198:179–82. doi: 10.1007/BF00328721 PMID:6394964

6. Brand A, MacCallum DM, Brown AJP, Gow NAR, Odds FC. Ectopic expression of *URA3* can influence the virulence phenotypes and proteome of *Candida albicans* but can be overcome by targeted reintegration of *URA3* at the *RPS10* locus. *Eukaryot Cell* (2004) 3:900–9. doi: 10.1128/EC.3.4.900-909.2004 PMID:15302823

7. Fonzi WA, Irwin MY. Isogenic strain construction and gene mapping in *Candida albicans*. *Genetics* (1993) 134:717–28. doi: NODOI PMID:8349105

8. Lo HJ, Köhler JR, Didomenico B, Loebenberg D, Cacciapuoti A, Fink GR. Nonfilamentous *C. albicans* mutants are avirulent. *Cell* (1997) 90:939–49. doi: 10.1016/S0092-8674(00)80358-X PMID:9298905

9. Liu H, Kohler J, Fink GR. Suppression of hyphal formation in *Candida albicans* by mutation of a *STE12* homolog. *Science* (1994) 266:1723–6. doi: 10.1126/science.7992058 PMID:7992058

10. Zheng X, Wang Y, Wang Y. Hgc1, a novel hypha-specific G1 cyclin-related protein regulates *Candida albicans* hyphal morphogenesis. *EMBO J* (2004) 23:1845–56. doi: 10.1038/sj.emboj.7600195 PMID:15071502

11. Bates S, MacCallum DM, Bertram G, Munro CA, Hughes HB, Buurman ET, et al. *Candida albicans* Pmr1p, a secretory pathway P-type Ca^2+^/Mn^2+^-ATPase, is required for glycosylation and virulence. *J Biol Chem* (2005) 280:23408–15. doi: 10.1074/jbc.M502162200 PMID:15843378

12. Hobson RP, Munro CA, Bates S, MacCallum DM, Cutler JE, Heinsbroek SE, et al. Loss of cell wall mannosylphosphate in *Candida albicans* does not influence macrophage recognition. *J Biol Chem* (2004) 279:39628–35. doi: 10.1074/jbc.M405003200 PMID:15271989

13. Mora-Montes HM, Bates S, Netea MG, Diaz-Jimenez DF, Lopez-Romero E, Zinker S, et al. Endoplasmic reticulum alpha-glycosidases of *Candida albicans* are required for N glycosylation, cell wall integrity, and normal host-fungus interaction. *Eukaryot Cell* (2007) 6:2184–93. doi: 10.1128/EC.00350-07 PMID:17933909

14. Munro CA, Bates S, Buurman ET, Hughes HB, Maccallum DM, Bertram G, et al. Mnt1p and Mnt2p of *Candida albicans* are partially redundant alpha-1,2-mannosyltransferases that participate in *O*-linked mannosylation and are required for adhesion and virulence. *J Biol Chem* (2005) 280:1051–60. doi: 10.1074/jbc.M411413200 PMID:15519997

15. Bulawa CE, Miller DW, Henry LK, Becker JM. Attenuated virulence of chitin-deficient mutants of *Candida albicans*. *Proc Natl Acad Sci U S A* (1995) 92:10570–4. doi: 10.1073/pnas.92.23.10570 PMID:7479842

16. Sundstrom P, Balish E, Allen CM. Essential role of the *Candida albicans* transglutaminase substrate, hyphal wall protein 1, in lethal oroesophageal candidiasis in immunodeficient mice. *J Infect Dis* (2002) 185:521–30. doi: 10.1086/338836 PMID:11865405

17. Bailey DA, Feldmann PJF, Bovey M, Gow NAR, Brown AJP. The *Candida albicans HYR1* gene, which is activated in response to hyphal development, belongs to a gene family encoding yeast cell wall proteins. *J Bacteriol* (1996) 178:5353–60. doi: 10.1128/jb.178.18.5353-5360.1996 PMID:8808922

18. Zhao X, Oh SH, Cheng G, Green CB, Nuessen JA, Yeater K, et al. *ALS3* and *ALS8* represent a single locus that encodes a *Candida albicans* adhesin; functional comparisons between Als3p and Als1p. *Microbiology* (2004) 150:2415–28. doi: 10.1099/mic.0.26943-0 PMID:15256583

19. De Boer AD, De Groot PWJ, Weindl G, Schaller M, Riedel D, Diez-Orejas R, et al. The *Candida albicans* cell wall protein Rhd3/Pga29 is abundant in the yeast form and contributes to virulence. *Yeast* (2010) 27:611–24. doi: 10.1002/yea.1790 PMID:20533408
